# Liposome Reconstitution and Modulation of Recombinant Prenylated Human Rac1 by GEFs, GDI1 and Pak1

**DOI:** 10.1371/journal.pone.0102425

**Published:** 2014-07-11

**Authors:** Si-Cai Zhang, Lothar Gremer, Henrike Heise, Petra Janning, Aliaksei Shymanets, Ion C. Cirstea, Eberhard Krause, Bernd Nürnberg, Mohammad Reza Ahmadian

**Affiliations:** 1 Institute of Biochemistry and Molecular Biology II, Medical Faculty of the Heinrich-Heine University, Düsseldorf, Germany; 2 Institute of Physical Biology, Heinrich-Heine University, Düsseldorf, Germany; 3 Institute of Complex Systems, ICS-6, Research Center Jülich GmbH, Jülich, Germany; 4 Department of Chemical Biology, Max-Planck Institute of Molecular Physiology, Dortmund, Germany; 5 Institute of Experimental and Clinical Pharmacology and Toxicology, Tübingen Medical School, Tübingen, Germany; 6 Leibniz Institute for Age Research, Jena, Germany; 7 Laboratory of Mass Spectrometry, Leibniz Institute of Molecular Pharmacology, Berlin, Germany; Institute of Molecular and Cell Biology (IMCB), Singapore

## Abstract

Small Rho GTPases are well known to regulate a variety of cellular processes by acting as molecular switches. The regulatory function of Rho GTPases is critically dependent on their posttranslational modification at the carboxyl terminus by isoprenylation and association with proper cellular membranes. Despite numerous studies, the mechanisms of recycling and functional integration of Rho GTPases at the biological membranes are largely unclear. In this study, prenylated human Rac1, a prominent member of the Rho family, was purified in large amount from baculovirus-infected *Spodoptera frugiperda* insect cells using a systematic detergent screening. In contrast to non-prenylated human Rac1 purified from *Escherichia coli*, prenylated Rac1 from insect cells was able to associate with synthetic liposomes and to bind Rho-specific guanine nucleotide dissociation inhibitor 1 (GDI1). Subsequent liposome reconstitution experiments revealed that GDI1 efficiently extracts Rac1 from liposomes preferentially in the inactive GDP-bound state. The extraction was prevented when Rac1 was activated to its GTP-bound state by Rac-specific guanine nucleotide exchange factors (GEFs), such as Vav2, Dbl, Tiam1, P-Rex1 and TrioN, and bound by the downstream effector Pak1. We found that dissociation of Rac1-GDP from its complex with GDI1 strongly correlated with two distinct activities of especially Dbl and Tiam1, including liposome association and the GDP/GTP exchange. Taken together, our results provided first detailed insights into the advantages of the *in vitro* liposome-based reconstitution system to study both the integration of the signal transducing protein complexes and the mechanisms of regulation and signaling of small GTPases at biological membranes.

## Introduction

The Rho family GTPases are known to play an important role in diverse cellular processes and progression of different diseases, such as cardiovascular diseases, developmental and neurological disorders, as well as in tumor invasion and metastasis [1]. Rho GTPases share two common functional characteristics, membrane anchorage and an on/off switch cycle [2], [3].

Subcellular localization of Rho GTPases to different cellular membranes is known to be critical for their biological activity. This is achieved by a hypervariable region (HVR) [4] and a lipid anchor in their C-terminal tail at a distinct cysteine residue in the CAAX motif (C is cysteine, A is any aliphatic amino acid, and X is any amino acid) [2], [5], [6], [7]. In addition to either geranylgeranylation or farnesylation at the CAAX motif, some members of the Rho family, such as Rac1, require the C-terminal polybasic region and palmitoylation, essential for plasma membrane targeting and interaction with multiple lipids [8], [9].

Rho GTPase function is dependent on the guanine nucleotide-binding (G) domain that contains the principle binding center for GDP and GTP and binds depending on its nucleotide-bound state various regulators and effectors [3]. Thus, membrane-associated Rho GTPases act, with some exceptions [10], as molecular switches by cycling between an inactive GDP-bound state and an active GTP-bound state [10]. This cycle underlies two critical intrinsic functions, the GDP-GTP exchange and GTP hydrolysis [10] and is controlled by at least three classes of regulatory proteins [3]: (i) Guanine nucleotide exchange factors (GEFs), especially those of the diffuse B-cell lymphoma (Dbl) family, which catalyze the exchange of GDP to GTP and activate the GTPase [11], [12]; ii) GTPase activating proteins (GAPs), which enhance the GTP hydrolysis and convey the GTPase in its inactive conformation [13], [14]; (iii) Guanine nucleotide dissociation inhibitors (GDIs), which bind to prenylated Rho GTPases and extract them from the membranes into the cytoplasm [15], [16], [17]. The formation of the active GTP-bound state of Rho GTPases is accompanied by a conformational change in two regions (known as switch I and II; [3] which provide a platform for the selective interaction with structurally and functionally diverse effectors, *e.g.* p21-activated kinase α (Pak1). This class of proteins activate a wide variety of downstream signaling cascades [18], [19], [20] thereby regulating many important physiological and pathophysiological processes in eukaryotic cells [21], [22].

The last decades in research of small GTPases under cell-free conditions were prevalently dominated by non-membranous systems such as soluble, mostly C-terminally truncated GTPases as well as shortened regulatory and effector proteins, mostly comprised of either the minimal catalytically active regulatory domains (GAPs, GEFs) or, in the case of effectors, the GTPase-binding domains (GBDs). Since the basic molecular mechanism of GTPase regulation and effector interaction is largely elucidated, it is in fact necessary now to move from these simplified soluble systems to more physiological and complex systems, *i.e.* multi-domain binding proteins acting on prenylated GTPases bound to the lipid membranes, the site at which they normally achieve their function in cells. A crucial prerequisite is, therefore, the availability of large quantities of purified, posttranslationally modified GTPases. Several different strategies have been developed to obtain lipid-modified proteins. It has been shown that Cdc42 purified from human platelets and insect cells can be extracted from the liposomes by RhoGDI1 (called here GDI1) [23], [24]. Rac1 alone was purified from insect cells by using detergents [25], [26]. Robbe *et al*. have purified prenylated Rac1 in complex with GDI1 that stabilized Rac1 in aqueous solution [27], [28]. A similar strategy was used for the purification and structural determination of the Cdc42⋅GDP⋅GDI1 complex as well as of RhoA [29], [30]. Ugolev *et al*. have used an enzymatic method to modify Rac1 *in vitro* by using geranylgeranyl transferase I [31]. They have shown that Rac1 dissociated from GDI1 by the cooperative action of RacGEFs and phosphatidylinositol (3, 4, 5)-trisphosphate (PIP3) containing liposomes. Gureasko *et al*. have directly attached Ras covalently to liposomes by using chemical cross linking [32]. The latter strategy avoids difficulties inherent in purifying lipid-modified proteins but is not useful for extraction experiments of membrane-bound GTPases using GDIs [27] or, alternatively, the δ subunit of phosphodiesterase (PDEδ) [33], [34], [35]. A large number of studies have utilized the advantage of a chemical ligation of a synthesized, lipidated C-terminal peptide with the purified G domain of different small GTPases [36], such as Rab proteins [37], [38], [39], K-Ras [35] and RhoA [17]. However, the question of how naturally modified, liposome-bound small GTPases, *e.g.* Rac1, interact with their regulators and effectors remains to be unveiled.

In this study we established a novel protocol for the extraction and purification of recombinant, prenylated, functionally active human Rac1 using a baculovirus-insect cell expression system and a detergent screening. Subsequently, *in vitro* liposome reconstitution studies were performed to gain insights into the Rac1 association with liposomes of different lipid compositions, and the extraction of Rac1 by GDI1. Rac1 extraction was prevented by a GEF-mediated Rac1 activation and Pak1 interaction.

## Materials and Methods

### Constructs

Human *Rac1* (GenBank accession no. NM_006908.4) was subcloned into pFastBacHTB vector (Invitrogen, Carlsbad, CA) and fused with an N-terminal hexa-histidine (6xHis) tag. For bacterial expression, full-length *Rac1* and *GDI1* (GenBank accession no. D13989) were cloned into pGEX-4T1 vector. DHPH constructs of human *Vav2* (aa 168–543), human *Dbl* (aa 498–825), *TrioN* (aa 1226–1535), murine *Tiam1* (aa 1033–1404), human *P-Rex1* (aa 34–415), and human *Pak1*-GBD (aa 57–141) have been reported before [11], [40].

### Antibodies, media and reagents

Anti-His-tag (mouse), anti-Rac (mouse), anti-E-cadherin (rabbit), anti-GAPDH (rabbit), anti-histone H3 (rabbit), anti-α-tubulin (rabbit), anti-rabbit IgG (goat), anti-mouse IgG conjugated with Alexa Fluor^®^ 488 (goat), anti-Rabbit IgG conjugated with Alexa Fluor^®^ 594 (goat) were purchased from Invitrogen (Oregon, USA); anti-mouse IgG was obtained from Dako (rabbit, California, USA). GDP and a non-hydrolyzable GTP analogue, guanosine 5′-[β,γ-imido]triphosphate (GppNHp), were obtained from Jena Bioscience GmbH (Jena, Germany). TC100 insect cell media, fetal bovine serum, antibiotics (penicillin and streptomycin) and 10% Pluronic F-68 were obtained from PAN-Biotech GmbH (Aidenbach, Germany). Phosphatidylserine (PS), Phosphatidylcholine (PC), phosphatidylethanolamine (PE) and sphingomyelin (SM), phosphatidylinositol 4,5-bisphosphate (PIP2), and Folch I and Folch III brain lipid extracts were purchased from Sigma-Aldrich (Munich, Germany). PIP_3_ is from Merck (Darmstadt, Germany). All other standard reagents, including detergents (Table S1 in File S1) were obtained from Carl Roth GmbH (Karlsruhe, Germany) or Merck-Millipore (Darmstadt, Germany).

### Baculoviruses and insect cell culture

Human *Rac1* gene subcloned into pFastBacHTB vector (Invitrogen, Carlsbad, CA) was transformed into DH10BAC strain. Agar plates containing kanamycin, gentamycin, tetracycline, X-gal and isopropyl-β-D-thiogalactoside were used to select recombinant *Rac1* clones. The *Rac1*-positive clones underwent two more purification steps before recombinant *Rac1* bacmid were extracted. The baculoviruses (passage 1) were generated by infecting *Sf*9 insect cells using recombinant *Rac1* bacmids. Viruses were ready to use for large scale Rac1 expression after two more amplification steps (passages 2 and 3).

*Sf*9 were cultured in TC-100 medium, containing 10% fetal bovine serum, penicillin, streptomycin and pluronic F-68 solution at 27°C. The titer of baculoviruses was determined by the ITCD_50_ method [41], [42]. The multiplicity of infection (MOI) and Rac1 expression time were optimized by infecting the *Sf*9 cells at different MOIs and different culture time points. Samples of infected cells (1 ml) were harvested; the cell pellets were lysed in Laemmli buffer, containing 60 mM Tris-HCl pH 6.8, 2% SDS, 10% glycerol, 5% β-mercaptoethanol, 0.01% bromophenol blue and analyzed by immunoblotting using an anti-His-tag antibody.

### Confocal laser scanning microscopy (cLSM)

Insect cells were fixed with acetone/methanol (1∶1) at 24 hours after seeding in 12 well plates. Cells were incubated first with antibodies against Rac1 and α-tubulin and then with secondary anti-mouse and anti-rabbit antibodies conjugated with Alexa Fluor^®^ 488 and Alexa Fluor^®^ 594 as well as DAPI (Danvers, USA). All steps were carried out at room temperature. Specimens were visualized and photographed using a confocal laser scanning microscope (LSM510META; Zeiss, Jena, Germany).

### Cell fractionation

*Sf*9 cells were harvested, resuspended in a buffer, containing 10 mM HEPES-NaOH pH 7.9, 1.5 mM MgCl_2_, 10 mM KCl, 0.5 mM dithiothreitol (DTT), 1 tablet EDTA-free protease inhibitors (Roche, Mannheim, Germany), and disrupted under detergent-free conditions using pre-chilled Dounce homogenizer for 20 strokes with a tight pestle. Disrupted cells were centrifuged at 300x*g* for 5 min at 4°C. The supernatant was removed and centrifuged at 50000x*g* for 2 h at 4°C to separate the membrane (pellet) and the cytosolic fractions (supernatant). The pellet, containing enriched nuclei, was resuspended in 0.25 mM sucrose, 10 mM MgCl_2_ and a cushion of 0.88 mM sucrose and 0.5 mM MgCl_2_ was laid over. This sample was centrifuged at 2800x*g* for 10 min at 4°C to obtain the nuclear pellet. Protein samples from different fractions were analyzed by immunoblotting. Anti-Rac1 antibody was used to detect distribution of recombinant human Rac1 in all the fractions. Antibodies against E-cadherin, GAPDH and Histone H3 were used as marker for membrane, cytoplasmic and nuclear fractions, respectively.

### Detergent screening

Eighteen different detergents (Table S1 in File S1) were used to extract Rac1 from the membrane fraction of *Sf*9 insect cells. Detergents were used at 20% (w/v) stock solution in buffer, containing 50 mM Tris-HCl pH 7.5, 100 mM NaCl, 2 mM MgCl_2_, 10% glycerol, 20 mM β-glycerolphosphate, 1 mM ortho-Na_3_VO_4_ and 1 tablet EDTA-free inhibitor cocktail. The detergents at 1% and 0.5% (w/v) final concentrations were added into the suspension of membrane fractions, containing recombinant human Rac1. The mixtures were incubated at room temperature for 30 min and centrifuged at 20000x*g* for 10 min. The pellets and small amounts of supernatants were collected for immunoblot analysis. Residual supernatants were used further for pull-down assays with glutathione S-transferase (GST)-GDI1.

### Thin layer chromatography

To check the lipid composition of the liposomes thin-layer chromatography was conducted using a thin layer chromatography plate (silica, 20×20 cm; Macherey-Nagel GmbH, Düren, Germany) and a chloroform/methanol/water/acetic acid (60∶50∶4∶1) as eluting solvent system. Lipids were detected by molybdophosphoric acid spray.

### Protein purification and nucleotide exchange

Large scale *Rac1* expression was conducted according to the established protocol described above. *Sf*9 insect cells were inoculated at a density of 1.5×10^6^ cells/ml under optimized MOI and culture time. Cells were resuspended in lysis buffer, containing 50 mM HEPES-NaOH pH 7.4, 150 mM NaCl, 2 mM β-mercaptoethanol, 5 mM MgCl_2_, 0.1 mM GDP, 10 mM imidazole and the optimized detergents according to the screening procedure described above. Cells were disrupted by sonication in ice-water mixture. Supernatants were collected by centrifugation and loaded on a Ni-NTA Superflow column (Qiagen, Hilden, Germany). High salt buffer (50 mM HEPES-NaOH pH 7.4, 150 mM NaCl, 2 mM β-mercaptoethanol, 5 mM MgCl_2_, 0.1 mM GDP, 10 mM imidazole, 350 mM KCl and 1 mM ATP) was used to remove impurities from the target proteins. Rac1 protein was eluted using an imidazole gradient ranging from 10 to 500 mM. The protein solution was concentrated and further purified on a Superdex 75 column (10/300 GL, GE-Healthcare, Uppsala, Sweden) with 50 mM HEPES-NaOH pH 7.4, 150 mM NaCl, 3 mM DTT, 5 mM MgCl_2_ and 0.5% (w/v) Na-cholate as buffer system. GppNHp-bound Rac1 proteins as well as human GDI1, RacGEFs, Pak1, full length and C-terminal truncated Rac1 proteins were prepared from *E. coli* as described previously [11], [43].

### Pull-down assay

GST-fused human GDI1 bound to glutathione beads was used to pull-down prenylated Rac1 from supernatants in the detergent screening procedure. Because of a detergent-induced nucleotide depletion of Rac1 it was important to determine the content of GDP-bound Rac1 proteins. The respective supernatants and beads were mixed and rotated at 4°C for 30 min. Samples were centrifuged at 500x*g* for 30 sec. Beads were washed three times using the buffers described above and containing the corresponding detergents. The beads and supernatant were analyzed by immunoblotting using anti-Rac antibody.

### Liposome preparation

Liposome assays were performed by mixing and incubating the liposomes and purified Rac1 proteins. The mixtures were incubated for different time points and centrifuged at different speeds to separate the liposome pellets and supernatants for optimizing the centrifuging force. The liposomes were prepared as described previously [44]. Briefly, a lipid mixture (194 µg), containing 39% (w/w) PE, 16% (w/w) PC, 36% (w/w) PS, 4% (w/w) SM, and 5% (w/w) PIP2 or PIP3, was dried using light nitrogen stream. Obtained lipid film was hydrated with 300 µl of a buffer, containing 30 mM HEPES-NaOH pH 7.4, 50 mM NaCl, 3 mM DTT, 5 mM MgCl_2_. Sonication (20 s with minimal power, 50% off and 50% on) was employed finally to form liposomes. Folch I and Folch III brain lipids extracts were prepared in methanol and chloroform at a concentration of 25 mg/ml. Folch I contains different phosphoinositides, PS, and cerebrosides in a ratio of 1∶5∶4 [45]. Folch III is composed of 80% PS, 10% PE, 5% cerebrosides and 5% unidentified membrane lipids [46]. Folch I or Folch III liposomes (250 µg, respectively) were prepared under the same conditions in 300 µl of the HEPES buffer. Liposomes with increasing PS or PC were prepared by either PS/PE or PC/PE to analyze lipid composition of Folch III. PC was used as control. Total lipids used for each liposome preparation were constantly 250 µg in 300 µl buffer.

## Results

### Subcellular localization of human Rac1 overexpressed in insect cells

The baculovirus-*Spodoptera frugiperda* (*Sf*9) insect cell expression system was used to express and purify human Rac1 in a prenylated form. In order to obtain optimal Rac1 expression, the tissue culture infectious dose 50 (TCID_50_) method was utilized to determine titers of the baculovirus stocks as described before [41], [42]. A striking characteristic of baculovirus-infected *Sf*9 cells is the so-called cytopathic effect, which is observed as a reduction of cell numbers and swollen cell size depending on the extent of infection as compared to the non-infected, highly confluent culture (Fig. S1A in File S1). *Sf*9 cells were next infected at different MOIs and culture time length. Increasing amounts of baculovirus resulted in a slight, dose-dependent increase in Rac1 expression with a peak around 36 and 48 h post-infection, especially at a MOI of 4 or 5 (Fig. S1B in File S1). Confocal imaging analysis revealed that human Rac1 is predominantly localized at the plasma membrane (Fig. S1C in File S1). Cell fractionation experiments showed that Rac1 was mainly found in the membrane and endoplasmic reticulum-enriched nuclear fractions (Fig. S1D in File S1). These data clearly show that human Rac1 produced in insect cells exhibits similar characteristics as compared to endogenous Rac1 in mammalian cells, such as mouse embryonic fibroblasts and HeLa [47], regarding its cellular distribution [48].

### Detergent extraction and purification of prenylated human Rac1 overexpressed in insect cells

**Figure 1:**
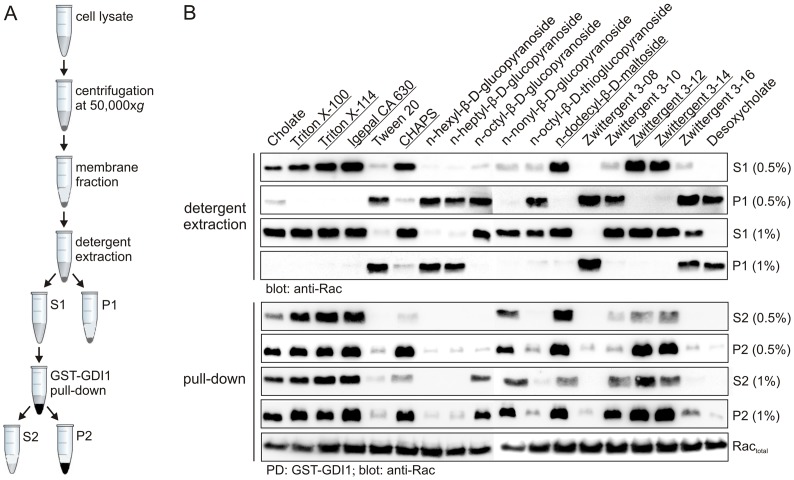
Detergent screening for optimal extraction of GDP-bound Rac1 from the insect cell membrane. (**A**) Schematic workflow for the isolation of insect cell membrane fraction, detergent extraction and pull-down assay using GST-GDI1. (**B**) Effects of eighteen various detergents on Rac1 extraction from the membrane fraction of insect cells (upper panel) and inspection of Rac1 prenylation *via* pull-down with GST-GDI1 (lower panel). Membrane fractions mixed with two different concentrations (0.5% and 1%) of the respective detergents (Table S1 in File S1) were incubated at room temperature for 30 min, separated in supernatants (S1) and pellets (P1) by centrifugation and immunoblotted using anti-Rac1 antibody. The Supernatants S1 were used in pull-down assays (PD) by using GST-GDI1, which selectively binds to the intact, nucleotide-bound Rac1. Resulted pellets (P2, corresponding to the GSH beads) and supernatant (S2) were visualized by anti-Rac1 antibody in immunoblots. Underlined detergents, especially CHAPS, showed the best properties in the extraction of GDP-bound Rac1 from the insect cell membranes.

An important issue to be considered for the extraction of nucleotide-bound, prenylated human Rac1 from *Sf*9 membrane fractions was the choice of an appropriate detergent. First attempts using deoxycholate and cholate as detergents were not successful. The former did not solubilize Rac1, while the latter did extract Rac1 but considerable amounts of extracted Rac1 proteins were depleted of their bound nucleotide (data not shown) indicating partial unfolding upon cholate treatment. It is of importance to note that a stoichiometric ratio of bound GDP is mandatory to avoid aggregation and precipitation of penylated Rac1, which means that the GDP-bound state must be monitored at every purification step, including detergent extraction from the cell membrane. Therefore, we tested sixteen additional detergents regarding their properties to extract fully functional Rac1 from the insect cell membrane fractions (Table S1 in File S1). Considering that the high amounts of detergent may also impair the quality of proteins, we used two different detergent concentrations (0.5% and 1% (w/v), respectively). Figure 1A illustrates a workflow with the corresponding steps of Rac1 extraction from the membrane and its pull-down by GST-GDI1. Seven detergents, *i.e.* Triton X-100, Triton X-114, Igepal CA 630, CHAPS, n-dodecyl-β-D-maltoside, Zwittergent 3–12 and Zwittergent 3–14 extracted similar amounts Rac1 from the membrane fraction at 0.5 and 1% concentrations (see supernatant fractions S1 in Fig. 1B, upper panel). In contrast, higher concentrations (1%) of cholate, n-octyl-β-D-glucopyranoside, n-nonyl-β-D-glucopyranoside, n-octyl-β-D-thioglucopyranoside, Zwittergent 3–10 and Zwittergent 3–16 were required to quantitatively extract Rac1 (Fig. 1B, upper panel). Tween 20, n-hexyl-β-D-glucopyranoside, n-heptyl-β-D-glucopyranoside and Zwittergent 3-08 were not useful at any concentrations (see pellet fractions P1 in Fig. 1B, upper panel).

After Rac1 was solubilized into the S1 fractions, purified GST fusion protein GST-GDI1 was employed to assess the functionality of soluble Rac1 in pull-down (PD) experiments, since only prenylated and GDP-bound Rac1 proteins are useful to study the RhoGDI interaction. From the seven detergents described above, CHAPS at 0.5% revealed the best property in extracting Rac1 from the insect cell membrane in its native, GDP-bound state (see P2 in Fig. 1B, lower panel). Almost all Rac1 proteins from the supernatant 1 (S1) were pulled down. In contrast, considerable amounts of Rac1 extracted by the other six detergents (Triton X-100, Triton X-114, Igepal CA 630, n-dodecyl-β-D-maltoside, Zwittergent 3–12 and Zwittergent 3–14) remained in the S2 fraction indicating that these Rac1 proteins are nucleotide-depleted or in incorrect conformation and thus inactive in binding to GST-GDI1 (Fig. 1B, lower panel).

Taken together, CHAPS displayed the two criteria required for further studies, namely to quantitatively solubilize Rac1 from insect cell membranes and to fully retain the GDI-binding activity of Rac1. Accordingly, 0.5% CHAPS was used to extract Rac1 from the membrane before successively applying the protein solution on two chromatography columns (Ni-NTA and size exclusion, respectively), in order to purify human Rac1 from insect cells (called from now Rac1^Ic^) at high quantities. Mass spectrometric analysis of intact Rac1^Ic^, compared to human Rac1 full length purified from *E. coli* (Rac1*^Ec^*), revealed a fully modified protein by geranylgeranylation with a modified most likely phosphorylated population (Fig. S2 in File S1).

### Human Rac1 purified from insect cells associates with liposomes

To analyze the membrane-binding properties of Rac1^Ic^ synthetic liposomes were prepared and sedimentation experiments were conducted according to the workflow illustrated in Figure S3A in File S1. To setup Rac1^Ic^ sedimentation by the liposomes various conditions were tested and optimized. One aspect was the incubation time after mixing Rac1^Ic^ with liposomes. Under the given conditions a weak binding of Rac1^Ic^ to the liposomes was observed, which was not significantly changed with increasing incubation time (Fig. S3B in File S1). We next analyzed the sedimentation force to avoid disruption of Rac1^Ic^-liposome interactions by incubating the samples for 30 min and using different centrifugation speeds to spin down the liposomes. Figure S3C in File S1 shows that increasing sedimentation force from 20,000x*g* to 60,000x*g* led to dissociation of Rac1^Ic^ from the liposomes suggesting that the sedimentation force should not exceed 20,000x*g*. In the next step we varied the ratio of Rac1 (1.5 µg) and liposomes (10 to 60 µl), and found out that as lower the ratio of Rac1^Ic^ to liposome is as larger are the Rac1^Ic^ amounts associated with the liposomes (Fig. S3D in File S1). The data clearly indicate that mixing of 1.5 µg Rac1^Ic^ with 20 µl liposomes for 20 min and centrifuging the sample at 20,000x*g* for 30 min provides optimal conditions for Rac1^Ic^ sedimentation with liposomes, which are used in following experiments.

The question of whether the lipid compositions of the liposomes may affect the liposome association of Rac1^Ic^ was next addressed using the optimized conditions described above. Data shown in Figure S3E in File S1 reveal that Rac1^Ic^-liposome interaction was only marginally affected upon depletion of the liposomes by individual phospholipids, especially PS and PIP2, by comparing the amounts of Rac1^Ic^ in the supernatants. As a control, we used Rac1*^Ec^*, which does not bind to the liposomes at all (Fig. S3E in File S1). Taken together, our data clearly demonstrate that Rac1^Ic^ is a lipidated protein and fulfills all criteria for the subsequent *in vitro* liposome reconstitution analysis.

### GDI1 interacts with and extracts Rac1^Ic^ from liposomes

**Figure 2:**
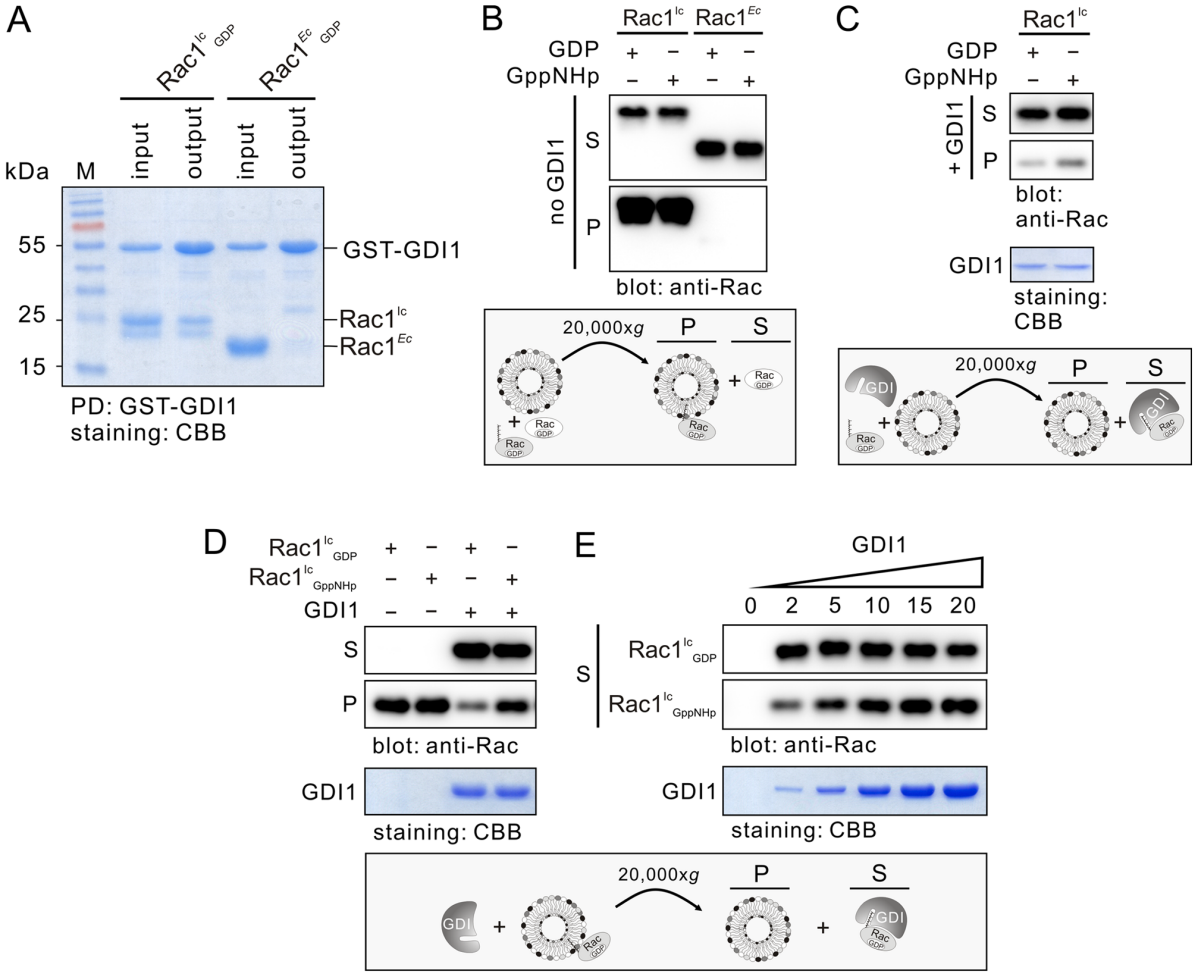
Nucleotide-independent extraction of Rac1^Ic^ from the liposomes by GDI1. (**A**) GST-GDI1 pull-down of Rac1^Ic^ but not of Rac1*^Ec^*. Input is the total mixture of beads and proteins, and output is the pull-down (PD). (**B**) Liposome binding of Rac1^Ic^ but not of Rac1*^Ec^*. In the liposome sedimentation assay, Rac1^Ic^ efficiently binds to liposomes in the absence of GDI1 and independent of whether it was loaded with GDP or GppNHp, a non-hydrolysable GTP analog. Rac1*^Ec^* failed to bind to liposomes under the same conditions. (**C**) Preferential binding Rac1^Ic^ to GDI1 than to liposomes. GDI1 binds to both GDP-bound and GppNHp-bound Rac1^Ic^ proteins and prevents their association with the liposomes. (**D**, **E**) GDI1 efficiently extracted GDP-bound Rac1^Ic^ from the liposomes and to a lower extend also Rac1^Ic^-GppNHp. Same amount of GDP-bound and GppNHp-bound forms of Rac1^Ic^ associated with the liposomes were prepared before incubation with 5-fold molar excess of GDI1 and sedimentation at 20,000x*g* (**D**). Using increasing molar excess of GDI1 (2-, 5-, 10-, 15- and 20-fold) showed that higher concentrations of GDI1 are required to extract Rac1^Ic^-GppNHp from the liposomes to supernatants in comparison to Rac1^Ic^-GDP (**E**). CBB, coomassie brilliant blue; *Ec*, *E. coli*; Ic, insect cells; P, liposome pellet; S, supernatant.

GDI1 is reported to solubilize Rac1 in living cells and inhibit GDP dissociation from Rac1 [23], for which a C-terminal geranylgeranylation of Rac1 is required [48]. Therefore, we examined the properties of Rac1^Ic^ interaction with liposomes and GDI1 by combining liposome sedimentation and GST-GDI1 pull-down assays. As controls, Rac1*^Ec^* was used. In addition, we prepared also inactive GDP-bound and stable active GppNHp-bound forms of the Rac1 proteins. GppNHp is a non-hydrolysable analogue of GTP. As shown in Figure 2A, GST-GDI1 pulled down only Rac1^Ic^ but neither Rac1*^Ec^*. Data obtained from the immunoblotting analysis of the supernatant and pellet fractions after liposome sedimentation showed that equal amounts of Rac1^Ic^ in GDP-bound and GppNHp-bound states were associated with the liposomes (Fig. 2B). Under these conditions, we did not observe any liposome binding of Rac1*^Ec^*. Association of Rac1^Ic^, but not Rac1*^Ec^*, with both GDI1 and liposomes clearly support the mass spectrometric data and proved that human Rac1 purified from insect cells is posttranslationally modified by geranylgeranylation.

GDI1 is known to extract inactive, GDP-bound Rho GTPases, such as Rac1, from membranes and hold them in a complex in the cytosol away from their sites of action at membranes [16], [23], [49], [50]. To test this issue on liposomes *in vitro* in more detail, we performed two types of experiments. In the first approach, liposomes, GDI1 and Rac proteins were mixed together and incubated for 20 min at room temperature. Subsequently, the samples were centrifuged at 20,000x*g* for 30 min, and the respective supernatants and liposome pellets were immunoblotted using an anti-Rac antibody. The majority of the Rac1^Ic^ proteins remained in the supernatant most likely in complex with GDI1 regardless of the nature of the bound nucleotide (Fig. 2C). Only a trace amount of Rac1^Ic^ protein, especially the GppNHp-bound form, was found in the liposome fraction. These data shows that GDI1 dominantly competes with the liposomes in binding Rac1^Ic^. In the second approach, we firstly prepared Rac1^Ic^-bound liposomes under the same condition as in the previous experiment but in the absence of GDI1, then mixed the sample with GDI1 and performed the liposome sedimentation experiment again. Figure 2D shows that GDI1 is able to extract Rac1^Ic^ from liposomes preferentially in the GDP-bound from. To further prove this observation we repeated these experiments using increasing concentrations of GDI1 of 2- to 20-fold molar excess above Rac1^Ic^, associated with liposomes. A 2-fold excess of GDI1 was sufficient to displace all “extractable” GDP-bound Rac1^Ic^ from the liposomes (Fig. 2E). In contrast, about 10-fold larger amounts of GDI1 were required to extract Rac1^Ic^-GppNHp from the liposomes (Figs. 2E). Taken together, our result clearly demonstrates that the majority of the membrane-bound Rac1 protein is extracted by GDI1 and remains GDI1 associated.

### Rac1^Ic^ activation counteracts its extraction from the liposomes by GDI1

**Figure 3:**
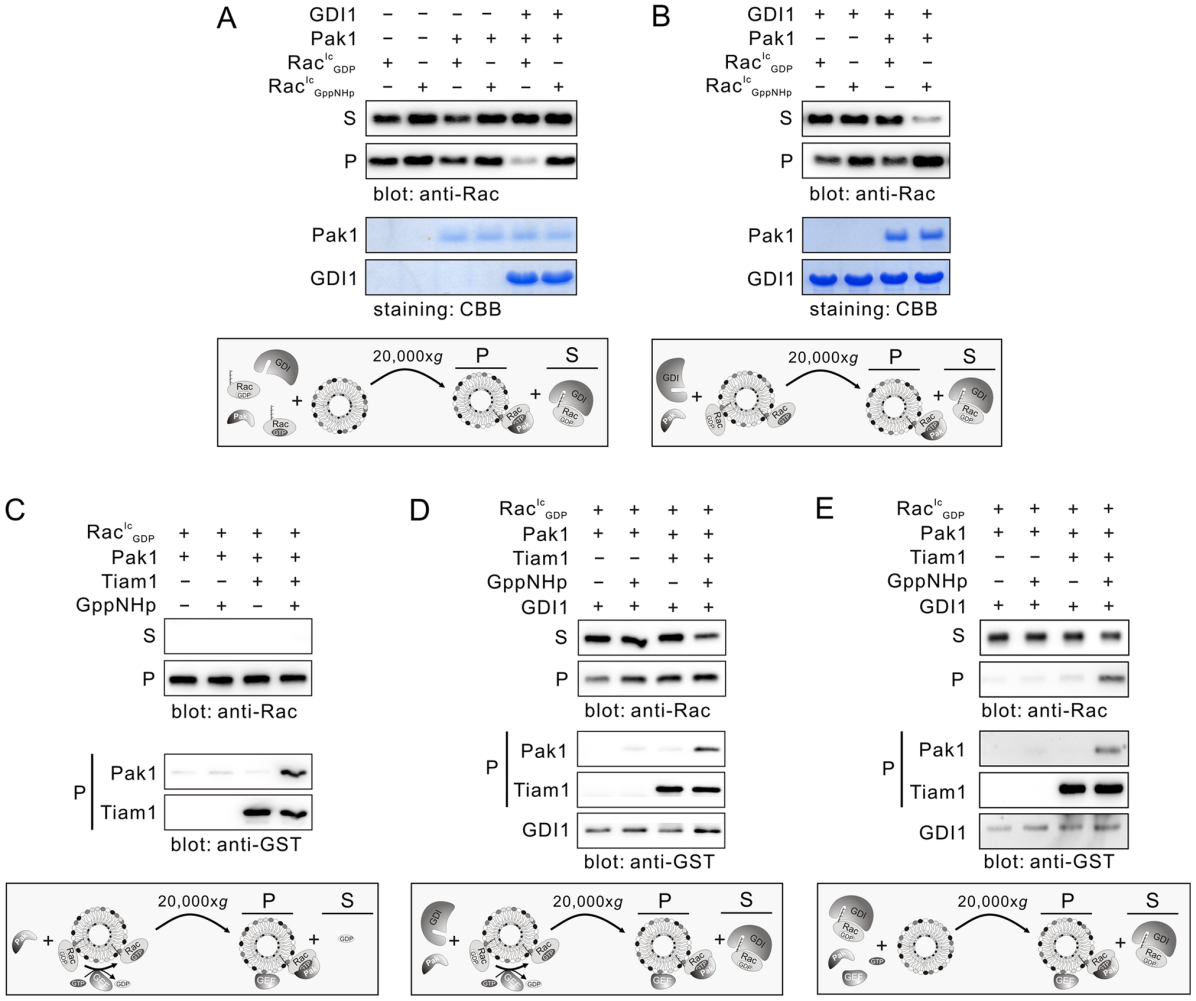
Pak1 binding to activated Rac1^Ic^ counteracting its extraction from the liposomes by GDI1. (**A**) Pak1 interferes with GDI1 binding to GppNHp-bound Rac1^Ic^ and potentiates Rac1^Ic^-GppNHp association with the liposomes. An excess amount of 20-fold of Pak1 was used in liposome sedimentation assay. (**B**) Competitive inhibition of the GDI1-mediated Rac1^Ic^-GppNHp extraction from the liposomes by Pak1. (**C**) Tiam1-mediated Rac1^Ic^ activation on the liposomes. Liposome-bound Rac1^Ic^-GDP was incubated with Pak1 in the presence or absence of Tiam1-DHPH and an excess of GppNHp. Rac1^Ic^ activation on liposome was evaluated by detecting Pak1 in the pellet, which is bound to Tiam1-activated Rac1^Ic^. (**D**) Tiam1-mediated Rac1^Ic^ activation on the liposomes in the presence of GDI1. (**E**) Partial displacement from the GDI1 complex, activation by Tiam1 and association of Rac1^Ic^ with liposomes and Pak1. CBB, coomassie brilliant blue; *Ec*, *E. coli*; Ic, insect cells; P, liposome pellet; S, supernatant.

We have shown above that GDI1 also binds Rac1^Ic^-GppNHp and extracts it from the liposomes (Fig. 2). To examine the interrelationship of this interaction, we conducted a series of liposome sedimentation experiments in the presence of the GTPase-binding domain (GBD) of Pak1 (called here Pak1). Mixing the Rac1^Ic^ proteins with liposomes, GDI1 and Pak1, respectively, revealed that Pak1 did neither influence the association of the GDP-bound Rac1^Ic^ proteins with the liposomes nor with GDI1 (Fig. 3A, lane 5). This data were comparable to the conditions when GDI1 was present and Pak1 absent (Fig. 2C, lane1). In contrast, Pak1 strongly counteracted a GDI1-mediated displacement of GppNHp-bound Rac1^Ic^ from the liposomes (Fig. 2C, lane2 and Fig. 3A, lane 6). In the next experiments we used liposome-associated Rac1^Ic^ proteins and showed that GDI1 extracted Rac1^Ic^ in both nucleotide-bound states from the liposomes in the absence of Pak1 (Fig. 3B, lane 1 and 2). Addition of Pak1 efficiently blocked GDI1-driven Rac1 extraction of GppNHp-bound Rac1^Ic^ from the liposomes (Fig. 3B, lane 4) but not that of the GDP-bound Rac1^Ic^ (Fig. 3B, lane 3). In agreement with the structural data [3], [28], [51], [52], our results suggest that Pak1 binding to the switch regions of active Rac1 competitively blocks the GDI1 association with the same regions of Rac1.

We next set out to analyze Rac1^Ic^ activation on the liposomes in the presence and in the absence of Pak1 and GDI1. We first prepared GDP-bound Rac1^Ic^ associated with liposomes, which were then incubated with free GppNHp and the DHPH domains of the RacGEF Tiam1 to accelerate the nucleotide exchange of Rac1^Ic^, leading to membrane bound Rac1^Ic^-GppNHp. GST-DHPH of Tiam1 as a minimal RacGEF protein contains the catalytic (Dbl homology or DH) and the lipid membrane binding (pleckstrin homology or PH) domains. GST-Pak1 was mixed in the samples as a marker for activated Rac1^Ic^ as it selectively binds to the active, GppNHp-bound state of Rac1 [53]. After incubation, the mixture was spun down and GST fusion in the pellet was visualized by immunobloting using anti-GST antibody. Results shown in Figure 3C revealed that Pak1 could be detected predominatantly in the liposome pellet only when both Tiam1 and GppNHp were present. In addition, DHPH was also detected in the pellet fraction. These data clearly indicate that Tiam1 DHPH was able to activate Rac1^Ic^ on the liposomes.

The next question we addressed was the ratio of soluble and liposome-bound Rac1 in the presence of GDI1, the RacGEF Tiam1 and the Rac effector Pak1. The majority of GDP-bound Rac1 appeared in complex with GDI1 (Fig. 3D, lane 1) indicating again that GDI1 efficiently extract Rac1^Ic^ from the liposomes. The picture slightly changed when the experiment was repeated also in the presence of Pak1 and GppNHp (Fig. 3D, lane 2) or Pak1 and Tiam1 (Fig. 3D, lane 3). There was, however, a significant limitation of the GDI-mediated Rac1 extraction from the liposomes observable when all components were in the sample (Fig. 3D, lane 4). This clearly demonstrates that a Tiam1-mediated exchange of the bound GDP for GppNHp resulted in the Rac1^Ic^-GppNHp-Pak1 complex formation on the liposomes as shown by Pak1 blotting (Fig. 3D, lane 4). This significantly blocked GDI1 association with and extraction of Rac1^Ic^ from the liposomes. This result suggests that Rac1 activation by Tiam1 largely counteracted the extraction of Rac1 from the liposomes by RhoGDI and shifts Rac1 towards a signaling-competent state.

However, the scenario substantially changes when Rac1^Ic^-GDP was not liposome-bound, like in the previous experiments, but in the complex with GDI (Fig. 3E). Under this condition, the presence of Tiam1, GppNHp and Pak1 was required to significantly release Rac1^Ic^-GDP from its GDI complex, to catalyze the nucleotide exchange by Tiam1 and to generate a liposome-bound Rac1^Ic^-GppNHp-Pak1 complex. This result clearly indicate that Tiam1 and Pak1 are certainly able to quantitatively displace the Rac1-GDP-GDI complex.

### Rac1^Ic^ activation by liposome-associating RacGEFs

**Figure 4:**
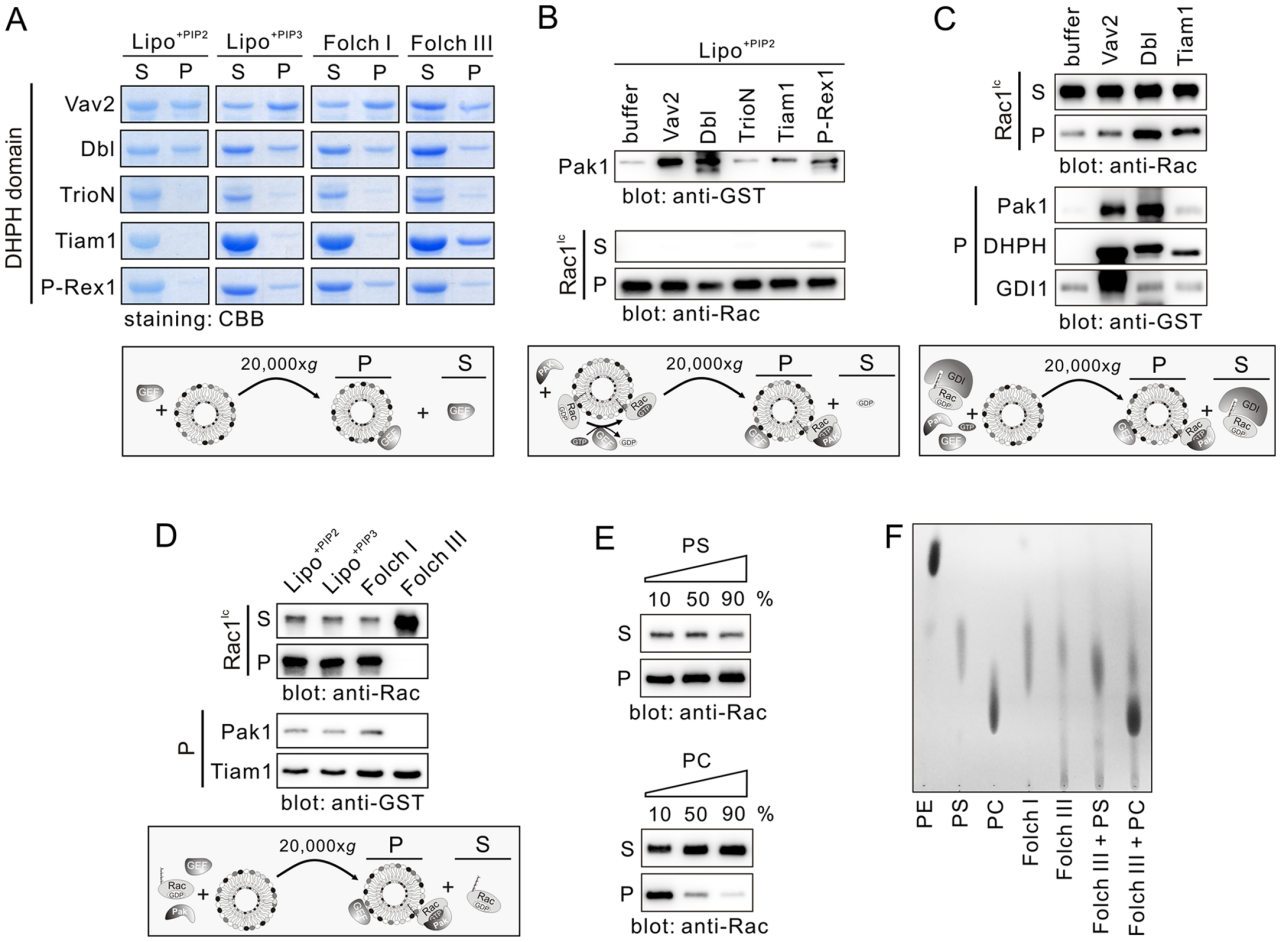
Rac1^Ic^ activation on liposomes by different Dbl family proteins and association with Pak1. (**A**) Differential binding of Vav2, Dbl, TrioN, Tiam1 and P-Rex1 to various liposomes. Lipo^+PIP2^ is composed of PE, PC, PS, SM and PIP2. In Lipo^+PIP3^, PIP2 is replaced by PIP3. (**B**) Efficient Rac1 activation by Dbl and Vav2 on Lipo^+PIP2^. (**C**) Displacement from the GDI1 complex, activation by GEFs and association of Rac1^Ic^ with liposomes and Pak1. (**D**) Tiam1 but not Rac1^Ic^ binding to Folch III. Rac1GDP, Pak1, Tiam1, GppNHp and different liposomes were preincubated, afterwards the liposome sedimentation assay was conducted. Rac1, PAKα and Tiam1 from the liposome pellet were detected by GST antibody. (**E**) Rac1^Ic^ repulsion by PC but not PS. Liposomes (PS and PE or PC and PE) at increasing amounts of PS and PC were sedimented after incubation with Rac1^Ic^. (F) Thin layer chromatography of Folch I and Folch III liposomes. Relative lipid content of Folch I and Folch III liposomes was analyzed by thin layer chromatography. PE, PS and PC as well as Folch III containing PS and PS were used as controls. CBB, coomassie brilliant blue; *Ec*, *E. coli*; Ic, insect cells; P, liposome pellet; S, supernatant.

The RhoGEFs of the Dbl family have been commonly implicated as lipid membrane binding modules [54]. The experiments described above have shown that Tiam1 DHPH activates liposome-bound Rac1, which can be atributed to the lipid membrane-binding PH domain [27], [55], [56]. Recently, we have shown that in addition to Tiam1 also Vav2, P-Rex1, Dbl, and TrioN are Rac1-specific GEFs [11]. These experiments have been performed under cell-free conditions in the absence of liposomes using nonprenylated Rac1 protein. Prior to the analysis of these Dbl proteins towards Rac1^Ic^, we analyzed their liposome-binding properties using the respective GST-DHPH proteins expressed and purified from *E. coli*. Therefore, we used four different types of liposomes, synthetic liposomes comprising in addition to PS, PC, PE and SM, either PIP2 (Lipo^+PIP2^) or PIP3 (Lipo^+PIP3^), as well liposomes derived from bovine brain type I and III Folch membrane lipids (see Materials and Methods). Figure 4A shows different liposome-binding capabilities of the five different Dbl proteins. Vav2, Dbl and P-Rex1 differently bound to all types of liposomes. TrioN and Tiam1 were hardly detected in Lipo^+PIP2^ but differently bound to the other liposomes. Interestingly, Tiam1 tightly bound to Folch III liposomes.

The capacity of the five Rac-specific Dbl proteins in the Rac1^Ic^ activation on the Lipo^+PIP2^ was next determined under the same conditions as described above (Figs. 3C and 3D). Consistent with their liposome binding pattern Vav2 and Dbl revealed the highest RacGEF activities shown as largest amount of Pak1 sedimented with activated, GppNHp-bound Rac1^Ic^ (Fig. 4B). P-Rex1, Tiam1 and TrioN activated Rac1^Ic^ to a lower extent (Fig. 4B), particularly TrioN, corresponding to their liposome binding capabilities (Fig. 4A). These data suggest that as stronger the respective Dbl protein interact with the liposomes as higher is its accessibility to Rac1. We next examined the capability of Vav2 and Dbl in displacing and activating Rac1^Ic^ from its complex with GDI1. Therefore, we mixed Rac1^Ic^-GDP-GDI1 with PIP2-containing liposomes, Pak1, GppNHp and the DHPH domains of Vav2, Dbl or Tiam1, respectively, and conducted liposome sedimentation. Dbl DHPH displaced and activated Rac1^Ic^ most efficiently as compared to Tiam1 and Vav2, which is visualized by a significant amount of Rac1^Ic^-GppNHp-Pak1 complex on the liposomes (Fig. 4C). Unexpectedly, this observation was not confirmed for Vav2, although a considerable amount of DHPH and Pak1 was sedimented with the liposomes (Fig. 4C). This result suggests that Dbl and Tiam1, but however not Vav2, contribute to displacement of Rac1 from the GDI complex by shifting the reaction towards active, GTP-bound Rac1 that is prepared for effector interaction and thus downstream signaling.

Tight Tiam1-binding to Folch III liposomes (Fig. 4A) prompted us to determine Tiam1 GEF activity on all four types of liposomes as we expected by far the highest Rac1^Ic^ activation on Folch III liposomes. Suprisingly, we obtained contrary results. In contrast to the other liposomes, on which Rac1^Ic^ was modestly activated by Tiam1, Folch III did not bind Rac1^Ic^ at all (Fig. 4D). As a consequence, Pak1 sedimentation could not be detected, although Tiam1 was presented on the Folch III liposomes (Fig. 4D). Folch III has been described to contain mainly PS (80%), and minor contents of PE (10%), cerebrosides (5%), and other unidentified membrane lipids (5%) [46]. In fact, we expected Rac1^Ic^, due to the positive electrostatics, immediately upstream of the prenylated cysteine 189 at its very C-terminus (^183^KKRKRKCLLL^192^), to bind tightly to the abundant, negatively charged PS moiety present in Folch III. However, synthetic liposomes and liposomes composed of Folch I also contain 50% PS (see Materials and Methods). Thus, we tested the effect of increasing PS concentrations in synthetic liposomes on the Rac1^Ic^ binding and used PC as a control. Interestingly, not increasing PS concentrations but PC repelled Rac1^Ic^ from associating with the liposomes (Fig. 4E) clearly supporting the existence of both an electrostatic attraction in the effective potential between Rac1^Ic^ and PS-containing liposomes and an electrostatic repulsion in the case of PC-containing liposomes. These data also suggest that Folch III may contain a different material that repel Rac1^Ic^ from the liposomes, which cannot be PS. Therefore, we analyzed the content of our liposomes by conducting a thin layer chromatography. Data shown in Figure 4F revealed Folch III indeed contains PS and not PC. There is a trace of lipids that are less polar than PS, which may be the cause for the Rac1^Ic^ repulsion. These data strongly suggest that Rac1 association with the membranes depends in addition to isoprenylation and accessory proteins also on local lipid composition.

## Discussion

The cell membrane is a platform for signal transduction through transmembrane receptors and membrane-associated proteins, including heterotrimeric G proteins and small GTPases of the Ras superfamily. These proteins are essentially dependent on posttranslational modifications by isoprenylation, palmitoylation or myristoylation to achieve their function [57], [58], [59]. In addition to studies of structural and chemical aspects of the individual proteins and components of signaling pathways, the new challenge is to investigate the influence of the lipid membrane surface environment on the temporal and spatial regulation of signaling events. One approach is the *in vitro* liposome reconstitution using purified proteins and synthetic liposomes. To this end prenylated GTPases are purified from tissues, eukaryotic cells, such as yeast, or they are synthesized by chemical ligation of unmodified GTPases from *E. coli* with a synthetic peptide harboring an isoprenyl moiety [36]. In this study, we used the baculovirus-insect cell expression system to express and purify recombinant human Rac1 in a prenylated form. This system has the advantage to express recombinant genes from any origin and produce considerable amounts of modified proteins [60]. Purification of postranslationally modified GTPases, such as prenylated Rac1, is challenging in a way that its native, nucelotide-bound form needs to be maintained if extracted from the cell membranes. In a comprehensive detergent screen we found that some detergents, *e.g.* CHAPS, quantitatively extracted human Rac1-GDP from the insect cell membranes as monitored by a RhoGDI pull-down assay. Mass spectrometry, liposome- and RhoGDI-binding revealed that human Rac1 purified form insect cells is, in contrast to that purified from *E. coli*, posttranslationally modified.

Similar to our data, GDI1 has been reported previously to bind to and extract both nucleotide-bound forms of Cdc42 from plasma membranes *in vitro* [23], [24]. Robbe and colleagues have shown that purified GDP-bound Rac1 from insect cells was dissociated from its complex with RhoGDI and associated with liposomes when the bound GDP was exchanged for GTP by depleting the bound Mg^2+^ by EDTA treatment [27]. Fewer studies were conducted by using prenylated Rac1 protein alone to elucidate its interaction with GDI1 and the liposomes: It has been shown that Rac1 purified from insect cell membrane fractions interact with artificial phagocyte membranes and that GDI1 counteracted this process [61]. This regulatory process is visualized in the present study in a direct way. We showed that GDI1 preferentially associates with the inactive, GDP-bound Rac1 and displaces it from the membrane as reported previously [50]. In addition, we found that a displacement of the active Rac1-GppNHp is also possible in spite of its low affinity for the GDI1. However, this does not take place if a Rac1 effector is in the proximity, as we showed for PAK1.

We have shown above that GDI1 also binds Rac1-GppNHp, consistent with early reports [62], and extracts it from the liposomes, although not as efficiently as Rac1-GDP (Fig. 2). One reason is that only a few residues of the four regions on Rac1 (amino acids or aa 29–42 of switch I, aa 62–68 of switch II, aa 91–108 of α-helix 3 and aa 187–189 at the C-terminus), which are in direct contact with GDI [3], determine the specificity of the interaction of Rac-GDP with GDI [28], [63]. Interestingly, conserved residues, such as Val36 and Asp38 of switch I, and Arg68, Tyr66, Leu69 and Leu72 of switch II, do not only contribute to the interaction with the GDI, but also to the interaction with GAPs and effectors. Under this environmental condition on the surface of the plasma membrane GDI does probably not undergo any interaction with Rac1-GTP because it, as long as it is not switched off by GAPs, may preferentially exist in complex with various signal-transducing effectors, such as Pak1 [18], [20], [64]. This is exactly what we observed in this study when we mixed Rac1-GppNHp bound to liposomes with both GDI1 and Pak1. The latter binds Rac1 and blocks both, the accessibility of GDI1 and consequently Rac1-GppNHp extraction from the liposomes (Fig. 3B). As a downstream effector of Rac1, Pak1 and its GBD specifically and tightly bind to activated Rac1 [53], [65], [66], [67]. Based on these findings, we can propose that RhoGDIs may also displace GTP-bound Rho GTPases from the plasma membrane presuming there are no Rac-specific effectors or GAPs around.

Another issue to be discussed is the interrelationship between GDIs and GEFs in regulating members of the Rho family. Unlike three known human RhoGDIs [15], [16], the classical Dbl GEF family consists of 74 members in human [11]. They are characterized by a unique, catalytic DH domain often preceded by a pleckstrin homology (PH) domain indicating an essential and conserved function [11], [12], [54]. The PH domain has been implicated to serve multiple roles in signaling events anchoring GEFs to the membrane (*e.g. via* phosphoinositides) [54] and directing them towards their interacting GTPases which are already localized to the membrane [12]. In this regard, it is important to note that the bulk of the added GEFs remained in the soluble fraction in the presence of liposomes (Fig. 4A), which most likely is, except for Vav2 and Dbl, due to low binding affinity of the tandem PH domain for the lipid membrane. This clearly suggests that the GEF recruitment to the cell membrane underlays additional concerted mechanisms. One is that accessory binding domains, existing in some GEFs, may be necessary to promote membrane association. This includes an extra PH domain, e.g. in Tiam1 [68], a diacylglycerol binding C1 (protein kinase C conserved region 1) domain, e.g. in Vav proteins [69], or a Sec14 domain, *e.g.* in Dbl [70]. The other membrane-translocating mechanism involves adaptor proteins, such the G protein βγ subunits recruiting P-Rex1 [71] or the Arp2/3 complex recruiting Tiam1 [72], [73].

Moreover, we found that dissociation of Rac1-GDP from its complex with GDI1 strongly correlated with two distinct activities of the DHPH of especially Dbl and Tiam1, including PH-mediated association with liposomes and DH-mediated GDP/GppNHp exchange of Rac1 (Figs. 3E and 4C). This and the fact that the binding affinity of the DH domain for the GDP-bound Rho GTPase is in the lower micromolar range (Z. Guo, E. Amin, R. Dvorsky and M.R. Ahmadian, unpublished data) indicates that the Rho GTPase-GDI complex may not be as tight as it has been suggested previousely [17], [24]. However, future investigations will elucidate the displacement of Rac1 from the GDI complex in the presence of liposomes and RacGEFs, both of which participate in the mechanism of specific activation of Rac1 and are required for accurate downstream signaling. This study has just opened a new window into future mechanistic studies of Rac1 regulation and signaling.

## Supporting Information

File S1 Contains Table S1, Detergents used in this study. Supporting References to Table S1. Figure S1, Expression of human Rac1 in insect cells. Figure S2, Mass spectra of Rac1*^Ec^* (left panel) and Rac1^Ic^ (right panel) after deconvolution. Figure S3, Liposome preparation and sedimentation experiments using human Rac1^Ic^.
(PDF)
