# Supplementary material for: Liposome Reconstitution and Modulation of Recombinant Prenylated Human Rac1 by GEFs, GDI1 and Pak1
Source: PLoS One. 2014 Jul 11;9(7):e102425. doi: 10.1371/journal.pone.0102425 (PMC4094549; doi:10.1371/journal.pone.0102425)
Supplement: File S1 — Contains Table S1, Detergents used in this study. Supporting References to Table S1. Figure S1, Expression of human Rac1 in insect cells. Figure S2, Mass spectra of Rac1Ec (left panel) and Rac1Ic (right panel) after deconvolution. Figure S3, Liposome preparation and sedimentation experiments using human Rac1Ic. (PDF) [file pone.0102425.s001.pdf]

Supporting Information

Liposome reconstitution and modulation of recombinant prenylated human Rac1 by GEFs, GDI1 and Pak1

Si-Cai Zhang, Lothar Gremer, Henrike Heise, Petra Janning, Aliaksei Shymanets, Ion C. Cirstea, Eberhard Krause, Bernd Nürnberg, and Mohammad Reza Ahmadian
Institute of Biochemistry and Molecular Biology II, Medical Faculty of the Heinrich-Heine University, Düsseldorf, Germany.

Table S1. Detergents used in this study.

Detergent	MW(Da)	CMC[a]	Used concentration[b]	
Cholate	430	14 mM (0.6%)	11.6 mM (0.5%)	23.3 mM (1%)
Triton X-100	625	0.24 mM (0.015%)	8 mM (0.5%)	16 mM (1%)
Triton X-114	537	0.2 mM (0.011%)	9.3 mM (0.5%)	18.6 mM (1%)
Igepal CA 630	648	0.083 mM (0.0053%)	7.7 mM (0.5%)	15.4 mM (1%)
Tween 20	1228	0.06 mM (0.0073%)	4 mM (0.5%)	8.1 mM (1%)
CHAPS	615	6-10 mM (0.37-0.61%)	8.1 mM (0.5%)	16.2 mM (1%)
n-hexyl-β-D-glucopyranoside	264	250 mM (6.6%)	18.9 mM (0.5%)	37.8 mM (1%)
n-heptyl-β-D-glucopyranoside	278	79 mM (2.2%)	18 mM (0.5%)	36 mM (1%)
n-octyl-β-D-glucopyranoside	292	20-25 mM (0.58-0.73%)	17.1 mM (0.5%)	34.2 mM (1%)
n-nonyl-β-D-glucopyranoside	306	6.5 mM (0.2%)	16.3 mM (0.5%)	32.6 mM (1%)
n-octyl-β-D-thioglucopyranoside	308	9 mM (0.28%)	12.5 mM (0.39%)	25 mM (0.77%)
n-dodecyl-β-D-maltoside	511	0.1-0.6 mM (0.005-0.03%)	10 mM (0.51%)	20 mM (1.02%)
Zwittergent 3-08	280	330 mM (9.22%)	17.9 mM (0.5%)	35.8 mM (1%)
Zwittergent 3-10	308	25-40 mM (0.77-1.23%)	16.3 mM (0.5%)	32.5 mM (1%)
Zwittergent 3-12	336	2-4 mM (0.067-0.134%)	14.9 mM (0.5%)	29.8 mM (1%)
Zwittergent 3-14	392	0.1-0.4 mM (0.004-0.016%)	12.8 mM (0.5%)	25.5 mM (1%)
Zwittergent 3-16	392	0.01-0.06 mM (0.0004-0.002%)	12.8 mM (0.5%)	25.5 mM (1%)
Deoxycholate	415	2-6 mM (0.082%-0.25%)	12.1 mM (0.5%)	24.2 mM (1%)

[a] Critical micelle concentration; [b] detergent concentration used in this study has been reported previously [Supporting References 1-4].

Supporting References to Table S1

1. Bramley, P. M. & Taylor, R. F. (1985) The Solubilization of Carotenogenic Enzymes of Phycomyces-Blakesleeanus, *Biochim Biophys Acta.* **839**, 155-160.
2. Collins, R. F., Ford, R. C., Kitmitto, A., Olsen, R. O., Tonjum, T. & Derrick, J. P. (2003) Three-dimensional structure of the Neisseria meningitidis secretin PilQ determined from negative-stain transmission electron microscopy, *J Bacteriol.* **185**, 2611-2617.
3. Linke, D. (2009) Detergents: an overview, *Methods Enzymol.* **463**, 603-617.
4. Miskevich, F., Davis, A., Leeprapaiwong, P., Giganti, V., Kostic, N. M. & Angel, L. A. (2011) Metal complexes as artificial proteases in proteomics: A palladium(II) complex cleaves various proteins in solutions containing detergents, *J Inorg Biochem.* **105**, 675-683.

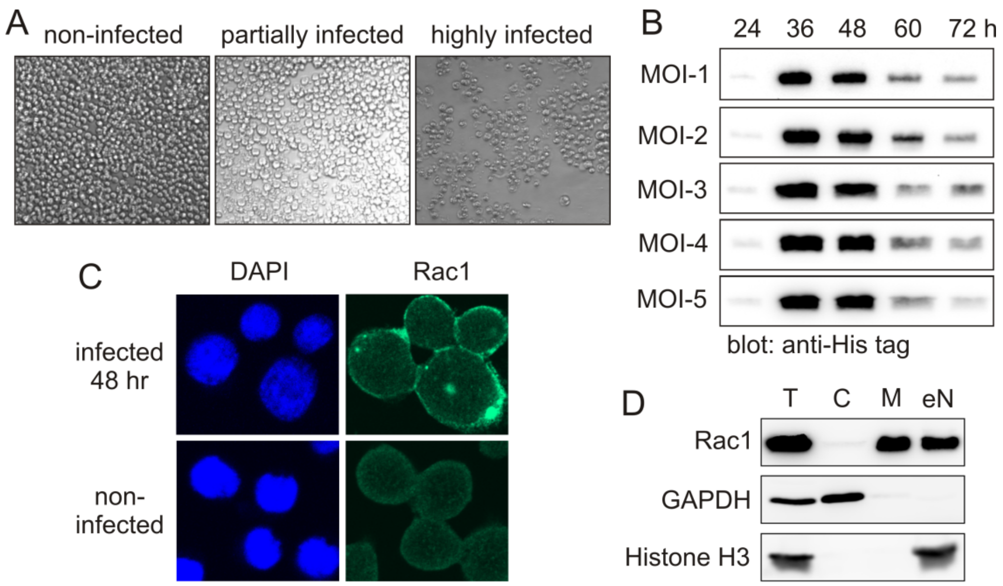

Figure S1. Expression of human Rac1 in insect cells. (**A**) The baculovirus cytopathic effects in *Sf*9 cells. Baculovirus-infected *Sf*9 cells are characterized by reduced cell numbers due to virus replication and subsequent cell death. (**B**) Rac1 expression in *Sf*9 cells. Insect cells are inoculated at different MOI (1 to 5) and harvested at different time points (24 to 72 h). Samples were analyzed by SDS-PAGE and immunoblotting using an anti-His tag antibody. (**C**) Rac1 localization at the plasma membrane of the *Sf*9 cells. Confocal laser scanning microscopy images of the insect cells depicting overexpressed Rac1 using anti-Rac1 antibody and nuclear staining using DAPI. Non-infected cells were used as control. (**D**) Subcellular fractionation of Rac1. *Sf*9 cells were fractionated by differential centrifugation into cytoplasmic (C), membrane (M) and enriched nuclear (eN) fractions. Total cell lysate (T) was used as control. Rac1 as well as GAPDH and histone H3 as markers were analyzed by SDS-PAGE and immunoblot analysis.

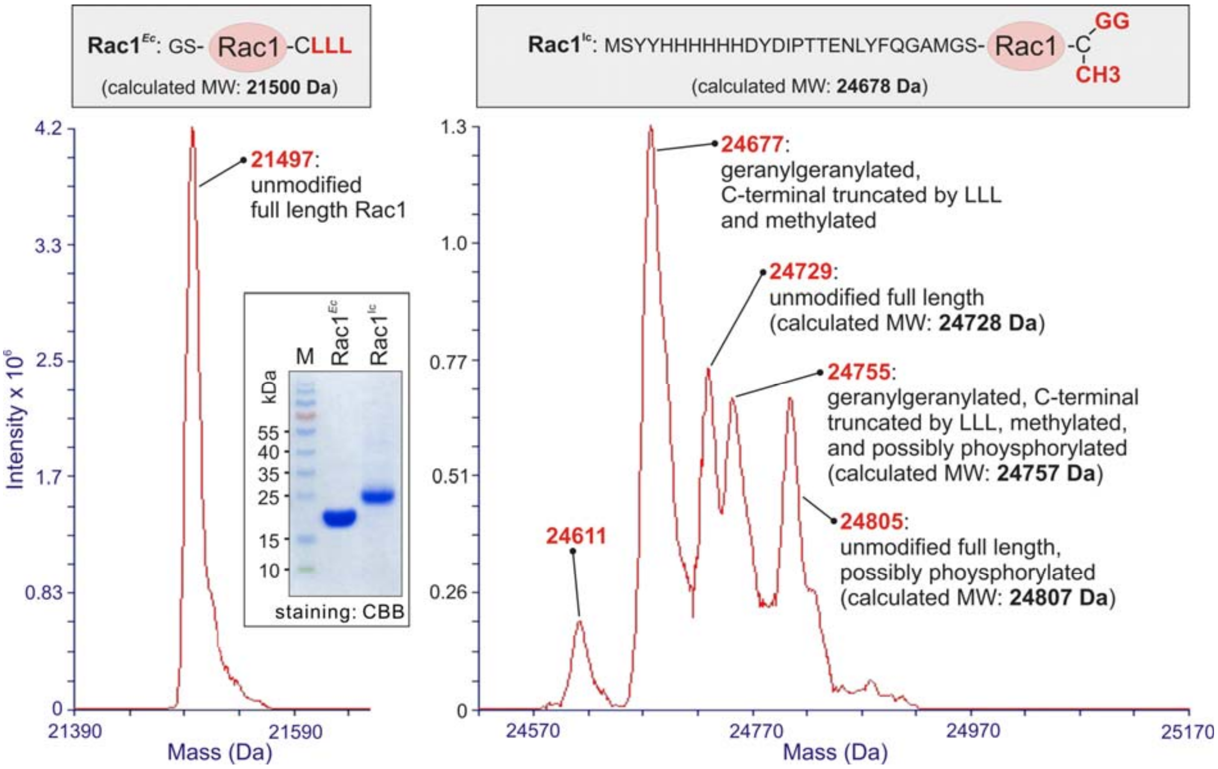

Figure S2. Mass spectra of Rac1*Ec* (left panel) and Rac1Ic (right panel) after deconvolution. Full length Rac1 proteins from *E. coli* and Rac1 from *Sf*9 insect cells were dissolved in 50% (v/v) acetonitrile and 0.2% (v/v) formic acid at a final concentration of 2 mg/ml. Proteins were subjected to a C4 HPLC column (Jupiter C4, 5 µm, 300 Å, 150 mm x 2 mm, Phenomenex, Aschaffenburg, Germany) equilibrated with 20 % (v/v) acetonitrile and 0.1 % (v/v) formic acid. For HPLC separation following conditions were used: HPLC-system 1100 series (Agilent Technology, Waldbronn, Germany), a flow rate of 200 µl/min, eluent A: 0.1 % (v/v) formic acid in water, eluent B: 0.1 % (v/v) formic acid in acetonitrile, gradient conditions: 20 % B for 2 min, linear gradient up to 50 % B in 23 min, linear gradient up to 90 % B in 15 min, 90 % B for 10 min, reequilibration of the column. The HPLC-system was coupled on-line to an ion trap mass spectrometer (LTQ, Thermo Fisher Scientific, Germany) equipped with an electrospray ionization source. Full spectra were acquired using a mass-to-charge range of 700 to 2000. Obtained spectra were deconvoluted using the program package Promass (Thermo Fisher Scientific, Germany). Masses obtained from the respective spectra are described with respect to calculated molecular weights (MW). Note that Rac1Ic contains at its N-terminus a 6xHis tag and a thrombin cleavage site. The sodium dodecyl sulfate polyacrylamide gel electrophoresis (SDS-PAGE) in the inset shows the purity of the two analyzed Rac1 proteins. CBB, coomassie brilliant blue; CH3, methyl group; Da, Dalton; *Ec*, *E. coli*; Ic, insect cells; GG, geranylgeranyl moiety; LLL, the very C-terminal three leucines of human Rac1; M, molecular weight marker.

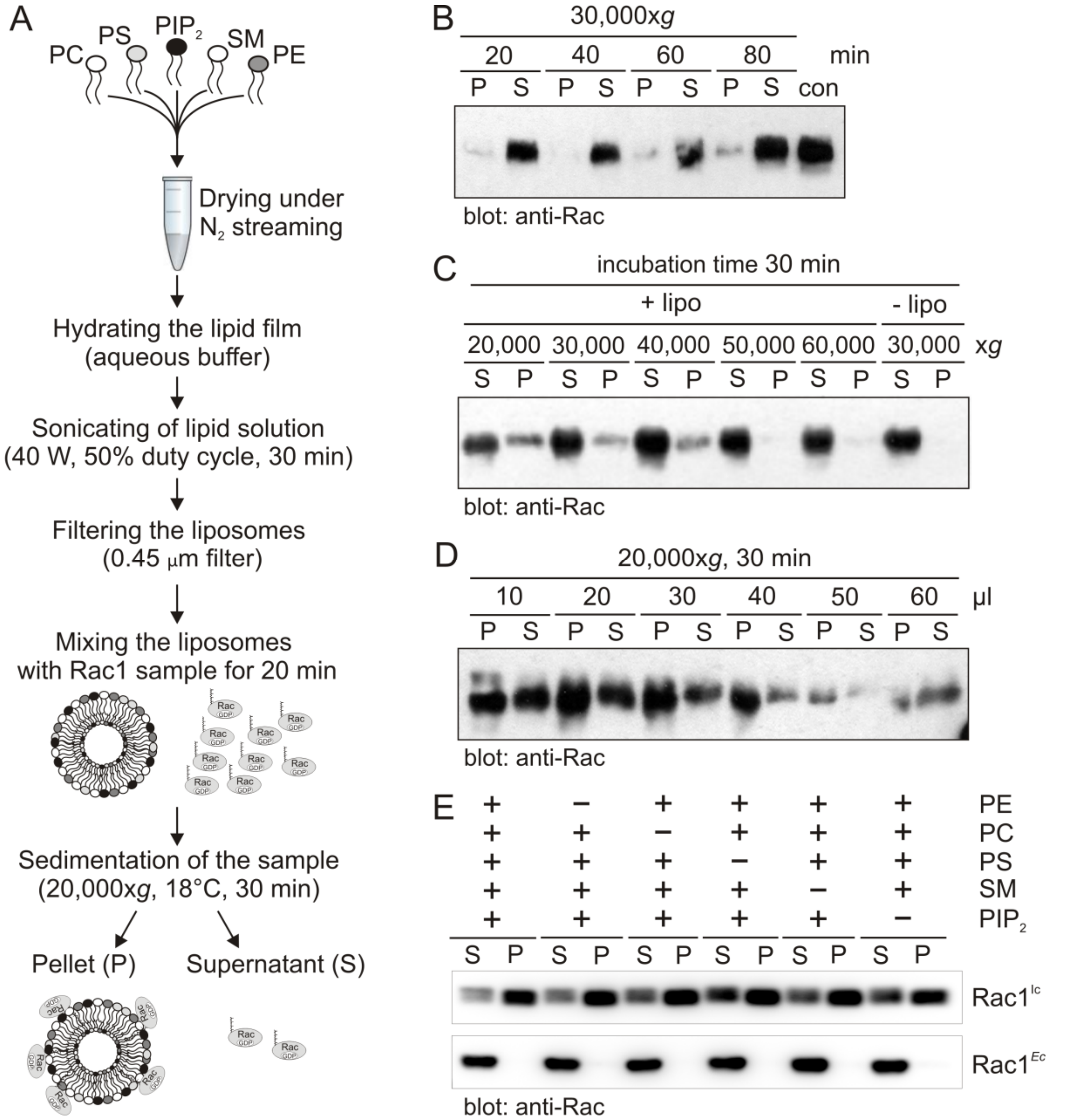

Figure S3. Liposome preparation and sedimentation experiments using human Rac1^{Ic}. (**A**) Schematic workflow of liposome formation using five different lipids, including phosphatidylcholine (PC), phosphatidylserine (PS), phosphatidylinositol 4,5-bisphosphate (PIP_2), sphingomyelin (SM), and phosphatidylethanolamine (PE). (**B**) The effect of different incubation time (20, 40, 60 and 80 min), (**C**) various centrifugation speeds (20,000, 30,000, 40,000, 50,000 and 60,000x*g*), (**D**) different amounts of liposomes (10, 20, 30, 40, 50 and 60 µl) and (**E**) distinct liposome composition on the association of Rac1^{Ic} (1.5 µg) with liposomes was analyzed using a liposome sedimentation assay. All samples were analyzed by immunoblotting using anti-Rac1 antibody. For the validation of specific interaction of Rac1^{Ic} with the liposomes, full length Rac1 purified from *E. coli* (Rac1^{Ec}) was used as control. Con, control; *Ec*, *E. coli*; Ic, insect cells; lipo; liposomes; P, liposome pellet; S, supernatant.
